# Too Hot to Handle: A Case of Esophageal Thermal Injury From Solid Food Ingestion

**DOI:** 10.1097/PG9.0000000000000286

**Published:** 2023-02-01

**Authors:** Tin Bo Nicholas Lam, Lauren Sussman, Benjamin Infantino

**Affiliations:** From the * Department of Pediatrics, Bernard and Millie Duker Children's Hospital, Albany Medical Center, Albany, NY; † Division of Pediatric Gastroenterology, Bernard and Millie Duker Children's Hospital, Albany Medical Center, Albany, NY

**Keywords:** esophageal injury, esophageal thermal injury, pediatric

## Abstract

Esophageal thermal injury (ETI) is an uncommon occurrence in both children and adults. Therefore, little is known about the diagnosis and clinical course of patients suffering from these injuries. We present the case of an 11-year-old female with macrocephaly capillary malformation syndrome and developmental delay suffering from ETI after ingestion of a piece of hot butternut squash. Endoscopy revealed linear, white plaques consistent with thermal burns. Management involved respiratory support, local and systemic analgesia, antibiotics, and nasogastric tube feedings. Our case highlights the nuances and differences in diagnosis, endoscopic findings, and management of ETI in a pediatric patient.

## INTRODUCTION

Esophageal thermal injury (ETI) is an uncommon occurrence in both children and adults. Thus, little is known about the diagnosis and clinical course of patients suffering from ETI. This case highlights the endoscopic findings and management of ETI in a pediatric patient.

## CASE REPORT

A nonverbal 11-year-old female with a history of macrocephaly capillary malformation syndrome and developmental delay was admitted for evaluation of poor oral intake after swallowing a piece of hot butternut squash at home. Her mother reported that the patient took a piece of butternut squash almost directly after it had been taken out of an oven running at 425°F. Within minutes, she showed progressive fussiness with eating, irritability, difficulties with vocalization, drooling, and emesis, all of which were atypical behaviors for her. She was immediately brought to the emergency department for evaluation.

**FIGURE 1.**
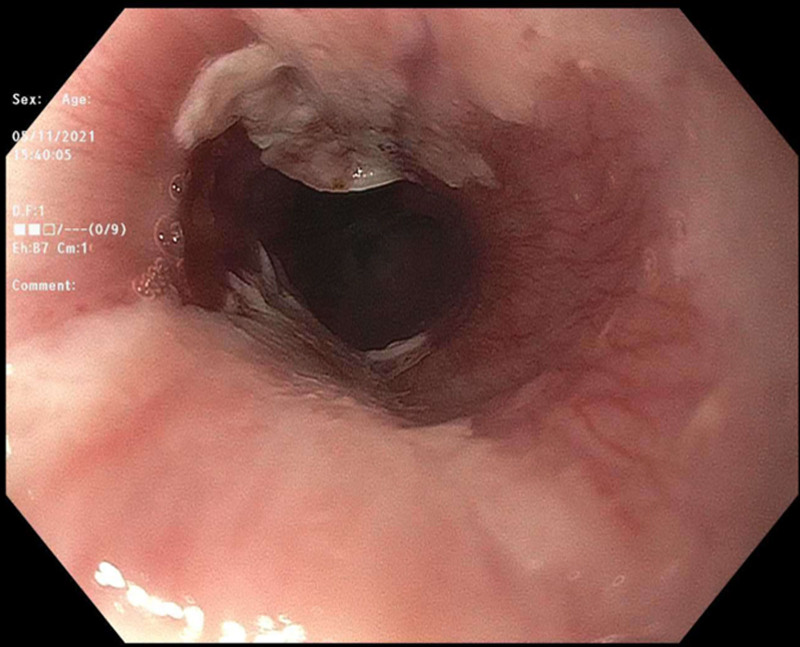
Endoscopic findings of esophageal inlet thermal injury (1).

**FIGURE 2.**
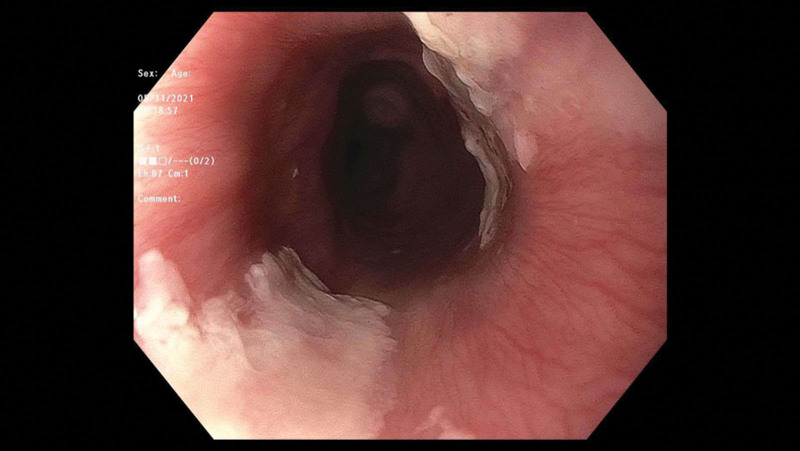
Endoscopic findings of esophageal inlet thermal injury (2).

On initial examination, she had normal vital signs and no obvious oral lesions or burns. Laboratory tests indicated no significant white blood cell count elevation concerning for infection and normal electrolytes. Otolaryngology service was consulted in emergency department and performed a bedside laryngoscopy that showed pharyngeal burns with significant mucosal burns at the base of the tongue and circumferentially around the esophageal inlet. They initially recommended treatment with ampicillin/sulbactam, dexamethasone for control of airway inflammation and a proton-pump inhibitor (PPI). Pediatric gastroenterology service was consulted, and upper endoscopy performed approximately 72 hours from initial injury showed opposing linear white plaques consistent with mucosal burns and superficial ulcers with overlying exudate at the esophageal inlet (Figs. 1 and 2).

Recommendation was made to add liquid sucralfate and ketorolac to her care to help her resume oral feedings due to refusal to take anything by mouth. Once tolerating soft solids consistently, she was discharged. Follow-up 10 months later via phone call revealed that she was continuing to tolerate soft solids without concern for dysphagia or regurgitation.

## DISCUSSION

ETIs are uncommon in both adult and pediatric populations, with ETIs secondary to solid food ingestion even rarer, especially in the pediatric population. A 2017 review of the literature on ETI by Lim et al (1) identified a total of 18 cases, of which only 6 had injuries after solid food ingestion. Furthermore, the youngest patient identified was 19 years old. Prevost et al (2) described a pediatric cases of ETI, in which a 16-year-old male consumed “hot mushroom tea”; however, this differed from our patient in that the ingestion was a liquid, not solid food. Unlike other patients with ETI, our patient did not present with the typical complaints of odynophagia, dysphagia, or chest pain. Due to our patient’s neurologic comorbidities and developmental delay, she was unable to verbalize her symptoms, making her presentation nonspecific.

Endoscopic findings in ETI patients with solid food ingestion differ from those of liquid ingestions. The characteristic candy-cane finding in hot liquid ingestions is rarely seen in solid food ingestions, likely due to the irregular shape and distribution of thermal contact as it passes through the esophagus (3,4). Though not commented upon in the final procedural report, review of our endoscopic images does not suggest evidence of this “candy-cane” characteristic finding. Instead, linear white plaques are observed, more consistent with continuous contact with a hot, solid object (Figs. 1 and 2).

Our patient received a combination of acid suppressive medication, corticosteroids, antibiotics, a cytoprotective agent, systemic analgesics, and enteral nutrition. In documented adult cases with similar extent of injury, medical management was limited to a combination of PPI and sucralfate, with initiation of parenteral nutrition (1). Our patient’s neurodevelopmental comorbidities arguably lead to reduced compliance, potentially necessitating more aggressive symptom control. Also, given this was a rare occurrence, guidelines for treatment were obscure in this setting. Corticosteroids were initiated by recommendation of otolaryngology to protect the airway by reducing edema. Antibiotic administration was likely a preventative measure. Unlike in adults, the principles of pediatric nutrition management favor early enteral feeding to support not only growth and development but recovery post injury.

Long-term surveillance endoscopy beyond initial presentation is uncommon and has been documented in one notable case by Kitajima et al, where the patient’s initial symptoms did not resolve with conservative treatment. This resulted in surgical excision of a severely strictured segment of esophagus, with subsequent ileocolonic interposition (5). In our case, strictures and adhesions were of concern due to the extent of damage. Thus far, long-term phone follow-up of our patient has shown no complications; however, repeat EGD or esophagram has not been performed. This, combined with a paucity of data in our patient population, makes long-term prognostication difficult.

## ACKNOWLEDGMENTS

Written consent was obtained from the patient’s parent in order to write and submit this case report.
